# Adipocere formation in biofilms as a first step in soft tissue preservation

**DOI:** 10.1038/s41598-022-14119-8

**Published:** 2022-06-16

**Authors:** Bastian Mähler, Kathrin Janssen, Mariam Tahoun, Frank Tomaschek, Rico Schellhorn, Christa E. Müller, Gabriele Bierbaum, Jes Rust

**Affiliations:** 1 Section Palaeontology, Institute of Geosciences, Rheinische Friedrich-Wilhelms Universität Bonn, 53115 Bonn, Germany; 2 Institute of Medical Microbiology, Immunology and Parasitology, Medical Faculty, Rheinische Friedrich-Wilhelms Universität, 53127 Bonn, Germany; 3 Pharmazeutisches Institut, Pharmazeutische und Medizinische Chemie, Rheinische Friedrich-Wilhelms-Universität Bonn, 53121 Bonn, Germany; 4 Section Geochemistry/Petrology, Institute of Geosciences, Rheinische Friedrich-Wilhelms-Universität Bonn, 53115 Bonn, Germany

**Keywords:** Palaeontology, Biofilms

## Abstract

The preservation of soft tissue in the fossil record is mostly due to the replacement of organic structures by minerals (e.g. calcite, aragonite or apatite) called pseudomorphs. In rare cases soft tissues were preserved by pyrite. We assume that adipocere, as the shaping component, might be a preliminary stage in the pyritisation of soft tissues under anaerobic conditions. Using high-performance liquid chromatography coupled to ultraviolet and mass spectrometric detection (HPLC–UV/MS) and confocal Raman spectroscopy (CRS) we were able to demonstrate the transformation of the hepatopancreas (digestive gland) of the crayfish *Cambarellus diminutus* [Hobbs 1945] into adipocere within only 9 days, just inside a biofilm. Microorganisms (bacteria and fungi) which were responsible for the biofilm (*Sphaerotilus* [Kutzig 1833] and *Pluteus* [Fries 1857]) and maybe the adipocere formation (*Clostridium* [Prazmowski 1880]) were detected by 16S rRNA gene amplicon sequencing. Furthermore, micro-computed tomography (µ-CT) analyses revealed a precipitation of calcite and further showed that in animals with biofilm formation calcite precipitates in finer grained crystals than in individuals without biofilm formation, and that the precipitates were denser and replicated the structures of the cuticles better than the coarse precipitates.

## Introduction

In aquatic environments, dead organisms are often covered by biofilms^1^. The effects of microbial activity on fossilisation processes are still being investigated. Taphonomic studies under laboratory conditions revealed various pathways how bacteria can influence the decomposition and lead to preservation^2,3^. The preservation of soft tissues (e.g. muscles) occurs in form of pseudomorphs, in which the original muscle tissue is replaced by calcium phosphate (CaPO_4_)^2–5^, or calcite^6^. In a recent experiment, the midbrain of a frog, that had been placed on a microbial mat was replaced by calcium carbonate (CaCO_3_) after 1.5 years^7^. In other studies, the hepatopancreases (digestive glands) of the shrimps *Crangon crangon* [Linnaeus 1758] and *Neogonodactylus oerstedii* [Hansen 1895] were mineralised by CaPO_4_^2,4,5^. Briggs and Kear^4^ even assumed that the mineralisation was initiated in the hepatopancreas. Taphonomic experiments have shown that these mineralisation processes are early diagenetic and dynamic, because tissue mineralised by CaPO_4_ can be covered by CaCO_3_ crystals if the pH switches to more alkaline conditions^2,8^. In addition, the pH in a decaying carcass might vary in different parts of the organism and result in the precipitation of various minerals (e.g. apatite, calcite, aragonite) in the same carcass^8^ or lead to the dissolution of minerals (e.g. amorphous calcium carbonate)^6^. For example, the fossils of the crustacean-like specimen *Dollocaris ingens* [Van Straelen 1924] from the Jurassic Konservat-Lagerstätte of La Voulte-sur-Rhône in France show a variation of different minerals inside the body cavity^9^.

In this study we describe the transformation of the hepatopancreas of *Cambarellus diminutus* [Hobbs 1945] into adipocere inside a biofilm. Adipocere is the result of incomplete hydrolysis of fat in animal tissue by bacteria under mainly anaerobic conditions^10^. Caused by the ability of adipocere to slow down or inhibit decay processes^11^ it has been suggested as a key component in the outstanding preservation of fossils in Konservat-Lagerstätten like Messel, Holzmaden^12^ or Solnhofen^13^. It is also assumed, that adipocere formation preceded the phosphatization of insects discovered at Quercy (France) as a shaping component^14^. Berner^15^ as well hypothesized, that well-preserved fossils in calcium carbonate (CaCO_3_) concretions may have formed originally as adipocere, which was later converted into CaCO_3_.

## Materials and methods

Individuals of the extant crayfish *Cambarellus diminutus* [Hobbs 1945] were taken from a breeding tank community raised in our lab. The animals were kept in 54 L tanks of 60 × 30 × 30 cm in size, at a constant water temperature of 28 °C. Tanks were filled with pipe water and fortified with “Biotopol C” water conditioner (JBL, GmbH & Co. KG, Neuhofen, Germany) to neutralise zinc (Zn) and lead (Pb) and to remove chlorine (Cl) and bind copper (Cu). The crayfish were fed with nothing but “Crab Natural” (Sera, GmbH, Heinsberg, Germany), a main food for crayfish (ingredients can be found in Supplement 1).

Thirteen individuals with partly filled guts, were sacrificed by placing them in an atmosphere of carbon dioxide (CO_2_). Specimens were not dried before weighing them on a micro scale. Lengths were measured from the anterior tip of the cephalothorax to the end of the pleon without the telson (Supplementary Table S1). At the beginning of each experiment oxygen saturation and pH-value were measured with an oxygen probe, OXPB-11 and pH-meter, PCE-PHD 1 (both PCE Deutschland GmbH, Meschede, Germany). Images of decomposing crayfish were taken by an i-Phone 12 mini. Images have 4032 × 3024 pixel and 24 bit with an exposure time of 1/50 s. The hepatopancreases (digestive glands) of C4 and C8 were lying in tank water at room temperature and were photographed by using a stereomicroscope (Stemi 2000, Carl Zeiss Microscopy Deutschland GmbH, Oberkochen, Germany) combined with an iPhone 12 mini holding by a Gosky Universal Digiscoping Smartphone Adapter, FBA_QHAPO21 (Gosky-optics, USA). The image of the calcite conglomerate in Fig. 3 was photographed with a stereo-zoom-microscope (Axio Zoom. V16, Carl Zeiss Microscopy Deutschland, Oberkochen, Germany). Final figures were created by using Adobe Photoshop CS5 (Adobe, Dublin, Republic of Ireland) with 300 dpi.

### Experiment 1

The first experiment was conducted twice with first 3 and secondly 4 dead crayfish specimens (C1 to C7) that were placed on artificial sediment inside a 54 L tank (tank 1) filled with pipe water and fortified with “Biotopol C” water conditioner, under a constant water temperature of 28 °C for a duration of 9 days. The pH of the water was 8 and had an oxygen saturation of 8 mg/L. In order to obtain a suitable biofilm, a piece of the crab food “Crab Natural” was placed next to the dead individuals (Table 1). This was done because it was observed that food rings have the potential to induce biofilms. Individuals were photographed once per day.

**Table 1:** Setups of three different experiments.

	Experiment 1.1	Experiment 1.2	Experiment 2	Experiment 3
Type of water	Pipe water	Pipe water	Pipe water	Pipe water
Amount of water	54 L	54 L	54 L	54 L
Water conditioner	Biotopol C	Biotopol C	Biotopol C	Biotopol C
pH-value	8	8	8	8
Temperature	28 °C	28 °C	28 °C	28 °C
Oxygen saturation	8 mg/L	8 mg/L	8 mg/L	8 mg/L
Special ingredients	Food ring	Food ring	Ostracods	–
Sediment type	Artificial	Artificial	Artificial	Artificial
Conducted in:	Tank 1	Tank 1	Tank 1	Tank 2
Number of individuals	3	4	3	3

After 9 days, crayfish remains were removed from the tank and analysed by using a stereomicroscope. In addition, crayfish remains were scanned by using a micro-CT device, if possible.

### Experiment 2

The second experiment was conducted with 3 crayfish individuals (C8 to C10) after experiment 1 inside the same tank (tank 1) and water under a constant water temperature of 28 °C, for a duration of 8 days. The pH of the water was 8 with an oxygen saturation of 8 mg/L (Table 1). The individuals were photographed once per day and after 8 days, crayfish remains were analysed by using a stereomicroscope. Contrary to experiment 1, the water was colonized by a large number of ostracods (Supplementary Fig. S1a,b), which had been introduced by the crab food used in experiment 1.

### Experiment 3

In the third experiment crayfish individuals C11 to C13 were placed inside another tank (tank 2) filled with pipe water, which was also fortified with “Biotopol C” water conditioner. The experiment was conducted under a constant water temperature of 28 °C for a duration of 7 days. The pH of the water was 8 with an oxygen saturation of 8 mg/L. The water was free from ostracods and no crab food was add to the experiment (Table 1). Afterwards, the crayfish remains were removed from the tank and analysed by stereomicroscopy.

At the beginning of each experiment carcasses were fully articulated and blue in colour. In addition, no symbiotic, parasitic or commensally organisms were found on the carcasses.

### DNA extraction

DNA was extracted from the biofilm with the ZymoBIOMICS DNA/RNA Miniprep Kit (Zymo Research, Irvine, USA). Biofilm samples of C1, C2 and C4 were transferred into ZR BashingBead™ Lysis Tubes (0.1 and 0.5 mm) with 750 µl ZymoBIOMICS Lysis solution. Bead beating was performed with a Precellys^®^ homogenizer (Bertin Technologies S.A.S., Montigny Le Bretonneux, FR), 6000×*g* for 30 s. Samples were subsequently processed according to the manufacturer’s instructions. DNA was eluted in 50 µl DNase/RNase-free water and DNA concentration and quality was checked using a NanoDrop One/OneC Microvolume-UV/VIS-spectrophotometer (Thermo Fisher Scientific, Waltham, Massachusetts, USA).

### 16S rRNA gene amplicon sequencing

For 16S rRNA gene sequencing, the V4 variable region of the 16S rRNA gene sequence was amplified with the specific 16S primers of 16S-515F (GTG CCA GCM GCC GCG GTA A) and 16S-806R (GGA CTA CVS GGG TAT CTA AT)^16^. Fungal ITS-region was amplified with specific ITS-primers (for: CTT GGT CAT TTA GAG GAA GTA A rev: GCT GCG TTC TTC ATC GAT GC). The PCR reaction was performed as a single-step PCR with the HotStarTaq Plus Master Mix Kit (Qiagen, USA) including an initial denaturation at 95 °C for 5 min, followed by 30–35 cycles of 95 °C for 30 s, 53 °C for 40 s, and 72 °C for 1 min, with a final elongation step at 72 °C for 10 min. Paired end sequencing (bTEFAP^®^) was performed by MR DNA (http://www.mrdnalab.com, Shallowater, TX, USA) on a MiSeq following the manufacturer’s guidelines^17^. Raw sequence data was processed via the QIIME2 pipeline^18^ with default parameters unless otherwise noted. DADA2 pipeline was used for sequence quality control, denoising and chimeric filtering^19^. Taxonomy classification of the final bacterial ASVs (amplicon sequencing variant), clustered at 99% identity, was performed with a naive Bayesian classifier which was trained against SILVA database release 138 especially for 515F/806R rRNA region^20,21^. ASVs of fungal composition analysis were aligned to a curated database derived from NCBI which was performed by the sequencing facility. For prediction of metabolic characteristics of the bacteria, the sequences were taxonomically classified with the Greengenes database^22^. The allocation of the phenotypes was then performed with BugBase^23^.

All raw sequence data related to this study are deposited in the European Nucleotide Archive (ENA) (European Bioinformatics Institute, EMBL-EBI) database a collaboration partner of the International Nucleotide Sequence Database (INSDC), [Study-Accession Number: PRJEB43756].

### Micro-computed tomography (µ-CT)

The propodus of the right chela of sample C4 and the complete carcasses of sample C1, C2 and C6 were removed from the tank and were scanned by using a phoenix|x-ray v|tomex s 240 micro-computed-tomography (µ-CT) scanner (GE Measurement and Control, Wunstorf, Germany) located at the Institute of Geosciences of the University of Bonn. The data set has a resolution of 12.66 µm; the scans were carried out at 80 kV and 100 µA. Three frames per projection were acquired by a timing of 500 ms for a total of 1000 projections. The CT data were processed using the software VG Studio Max 3.2 (Volume Graphics, Heidelberg, Germany) and Avizo 8.1 (Thermo Fisher Scientific, Schwerte, Germany) to reconstruct and visualize the precipitated crystal clusters inside the specimens and specimen remains.

### Confocal Raman spectroscopy (CRS)

*Cambarellus diminutus* [Hobbs 1945] hepatopancreas samples and reference materials as well as crystal clusters were analysed using a Horiba Scientific LabRam HR800 (located at the Institute of Geosciences, University of Bonn). Raman scattering was excited with a 784 nm diode laser as excitation source. The spectrometer was calibrated with the first-order Si Raman band at 520.7 cm^−1^. Data in the spectral region of 300 to 1800 cm^−1^ (hepatopancreas) and 100 to 1800 cm^-1^ (crystal clusters) were collected with a 100 × long working distance objective, a confocal hole size set to 1000 µm, spectrometer entrance slit size of 100 µm, and a grating of 600 grooves/mm. The exposure time was 42 min per window with 50 accumulations of 50 s for the hepatopancreas samples, and 4.2 min with 50 accumulations of 5 s for saturated fatty acid reference materials, respectively. The exposure time for crystal clusters was 2 min with 4 accumulations of 30 s.

### Scanning electron microscopy (SEM)

The right propodus and the dactylus, as well as a part of the hepatopancreas of sample C4 and crystal clusters were dissected and coated by a thin layer of gold with a cool sputter coater (Cressington Sputter Coater 108 manual, Tescan GmbH, Dortmund, Germany). Samples were subsequently scanned with an ‘environmental’ scanning electron microscope (SEM) unit (TESCAN VEGA 4 LMU) by using the SE detector at 20 keV. Images have 1536 × 1331 pixel and 16 bit. The working distance of each SEM-image can be found in the figure captions.

### High performance liquid chromatography coupled to ultraviolet and mass spectrometry detection (HPLC–UV/MS)

#### Materials and analytical conditions

Measurements were performed on an Agilent 1260 Infinity HPLC coupled to an Agilent Infinity Lab LC/MSD single quadrupole mass spectrometer with an electrospray ion (ESI) source and a diode array UV detector (DAD-UV, 200–600 nm, Agilent Technologies Germany GmbH & Co. KG, Waldbronn, Germany). Chromatographic separation was performed on an EC 50/3 Nucleodur C18 Gravity column, 3 μm (Macherey–Nagel, Dueren, Germany). Standard solutions of palmitic acid, oleic acid, and stearic acid (Sigma Aldrich Chemie GmbH, Taufkirchen, Germany) were prepared in a 1:1 solution of dichloromethane/acetonitrile, and known amounts were added to a sample for confirmation of retention times. A triglyceride mixture containing glyceryl trimyristate as the main component (Sigma Aldrich Chemie GmbH, Taufkirchen, Germany) was employed as a further standard. All solvents used were HPLC grade. Mobile phase A consisted of methanol with 2 mmol/l ammonium acetate, and mobile phase B consisted of water with 2 mmol/l ammonium acetate. The run started with 50% A and 50% B for 1 min, followed by a gradient that reached 100% of eluent A after 15 min. Then, the column was flushed for 10 min with 100% of mobile phase A followed by 50% A and 50% B for 5 min before starting the next run. Positive and negative full scan MS was obtained from 100 to 1000 m/z. The column temperature was set at 40 °C, the injection volume was 5 μl, and the flow rate was adjusted to 0.5 ml/min. Identification of the peaks was performed using the data analysis program of the OpenLab CDS 2.5 software (Agilent Technologies Germany GmbH & Co. KG, Waldbronn, Germany). The extracted ion chromatogram (EIC) was used to evaluate peak areas and to provide a semi-quantitative estimate of the detected compounds.

#### Extraction of adipocere components

A part of the hepatopancreas of sample C4, taken approximately 9 days post-mortem and hypothesised to be adipocere based on its visual appearance, was extracted and analysed by HPLC-(DAD-UV)-ESI-MS with the aim to identify the different fatty acid components of adipocere from the resulting chromatographic peaks and mass spectra. A 0.9 mg sample was extracted with 5 ml of dichloromethane to obtain the lipophilic constituents present. Then, aliquots were diluted 1:1 with acetonitrile and subsequently analysed by HPLC-(DAD-UV)-ESI-MS. Equal volumes of sample were measured with and without adding a mixture of palmitic acid, stearic acid and oleic acid as reference compounds (final concentration of each fatty acid was 1 µmol/l). This standard addition technique was used for confirmation of the presence of the individual fatty acids and to identify possible matrix effects affecting their retention times.

## Results

### General observations

#### Experiment 1

**Figure 1:**
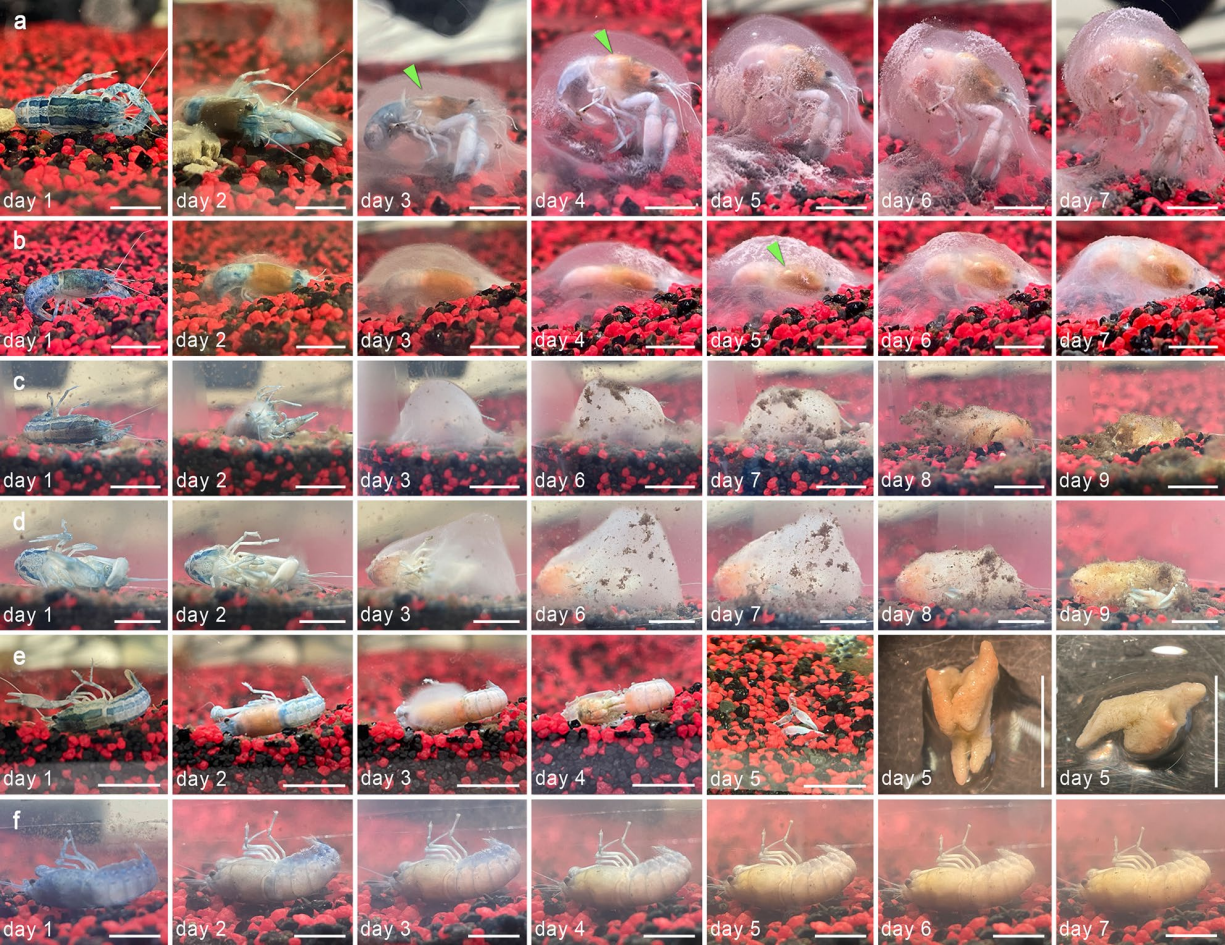
Decomposing crayfish individuals of three different experimental setups lying on artificial sediment at a constant water temperature of 28 °C. (**a**) Day 1 Dead articulated crayfish specimen C1, lying on its right body side in tank water. Day 2 Dead crayfish was moved by the development of a biofilm and the cephalothorax had changed its colouration from blue to red-brown. Day 3–7 Envelopment of the carcass by a biofilm and gas accumulation at the branchial area (green arrows), resulting in a “floating” carcass inside the biofilm. (**b**) Day 1 Dead articulated crayfish specimen C2, lying on its right body side in tank water. Day 2 Development of a biofilm around the cephalothorax, which had changed its colouration from blue to red-brown. Days 3–7 Envelopment of the carcass by a biofilm and gas accumulation at the branchial area (green arrow), resulting in a light “floating” carcass inside the biofilm. (**c**) Crayfish specimen C3 lying in tank water for a duration of nine days. Day 1 Dead articulated, blue crayfish lying on its right body side. Day 2 Cephalothorax had changed its colouration from blue to red-brown and is covered by a light, white biofilm. Day 3 The complete carcass was covered by a white biofilm and abdominal muscles had changed their colouration from white to pink. Days 6–9 Biofilm population by ostracods and its complete degradation. Day 9 Degraded cuticle of the pleon and some remains of the branchiae. (**d**) Crayfish specimen C4 in tank water for a duration of nine days. Day 1 Dead articulated, blue crayfish lying on its left body side. Day 2 Cephalothorax had changed its colouration from blue to red-brown and is covered by a light, white biofilm. Day 3 The complete carcass was covered by a white biofilm and abdominal muscles had changed their colouration from white to pink. Days 6–9 Biofilm population by ostracods and its degradation. Day 9 Remains of the biofilm and degraded cuticle. Chelipeds still intact and filled with pulpy muscles. (**e**) Crayfish specimen C8 in tank water for a duration of five days. Day 1 Dead articulated, blue crayfish lying on its left body side. Day 2 Cephalothorax had changed its colouration from blue to red. Day 3 The cephalothorax was covered by a white biofilm and abdominal muscles had changed their colouration form white to pink. Day 4 Carcass was populated by ostracods and the biofilm was completely degraded. Day 5 Only empty chelipeds and the hepatopancreas were left. (**f**) Crayfish specimen C11 lying in tank water for a duration of seven days. Days 1–7 Decomposing crayfish lying on its left bodyside. Cuticles of the cephalothorax and the pleon became light red and translucent. Muscles were pink in colour. Individuals were still articulated at day 7. All scale bars: 1 cm except the scale bars of the last two pictures in (**e**) which is 0.5 cm.

**Figure 2:**
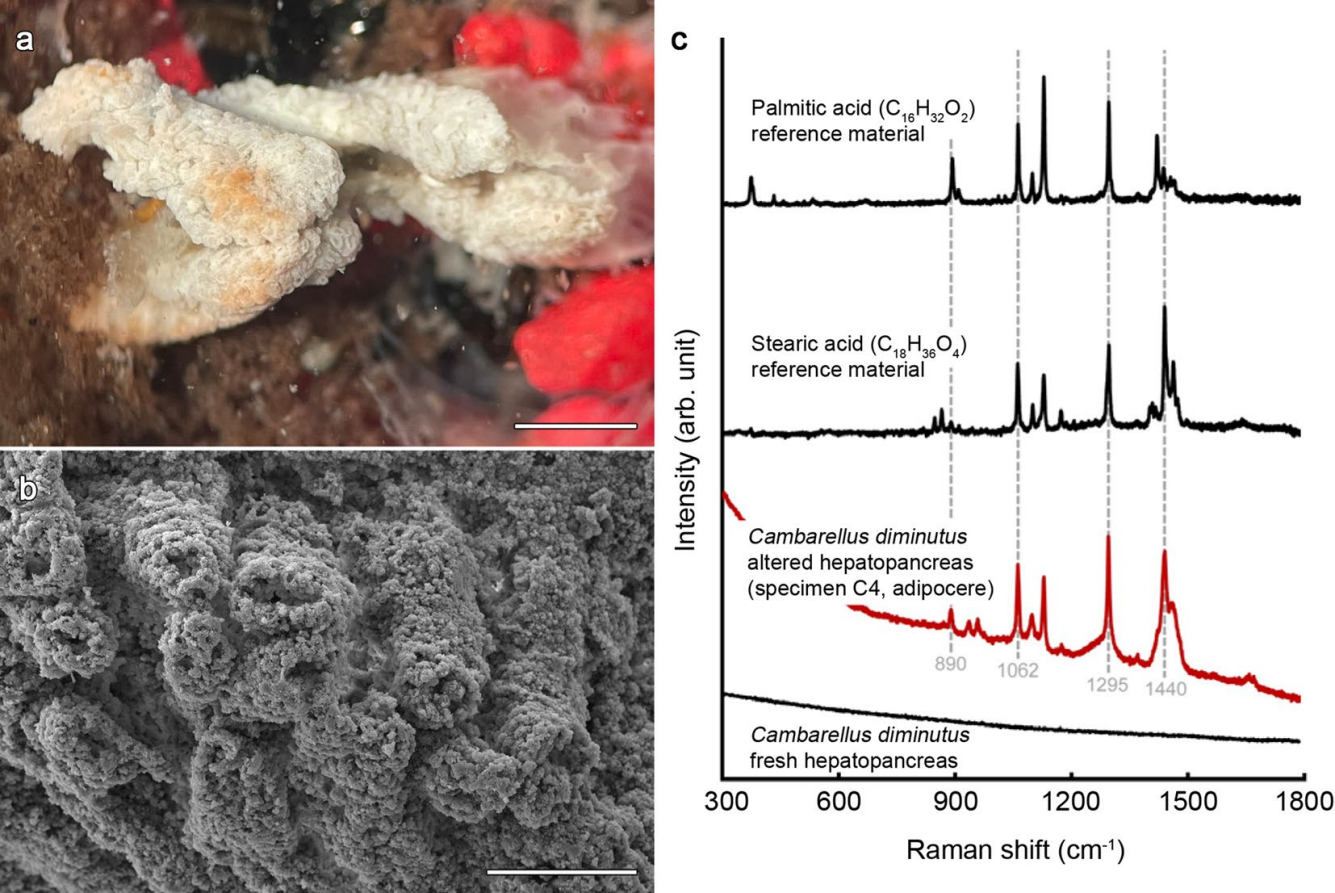
Images of the hepatopancreas (digestive gland) of crayfish specimen C4 after nine days in tank water covered by a biofilm and Raman spectra. (**a**) Stereomicroscopic image of the hepatopancreas in water. Scale bar 1 mm. (**b**) SEM-image of an enhanced part of the dried hepatopancreas [WD: 22.01 mm]. Scale bar 200 µm. (**c**) Representative Raman spectra of an altered *Cambarellus diminutus* hepatopancreas (specimen C4, adipocere), reference data for saturated fatty acids (stearic and palmitic acids), and the hepatopancreas of a freshly killed *C. diminutus*. Raman bands typical for saturated fatty acids have developed post‐mortem. *WD* working distance.

**Figure 3:**
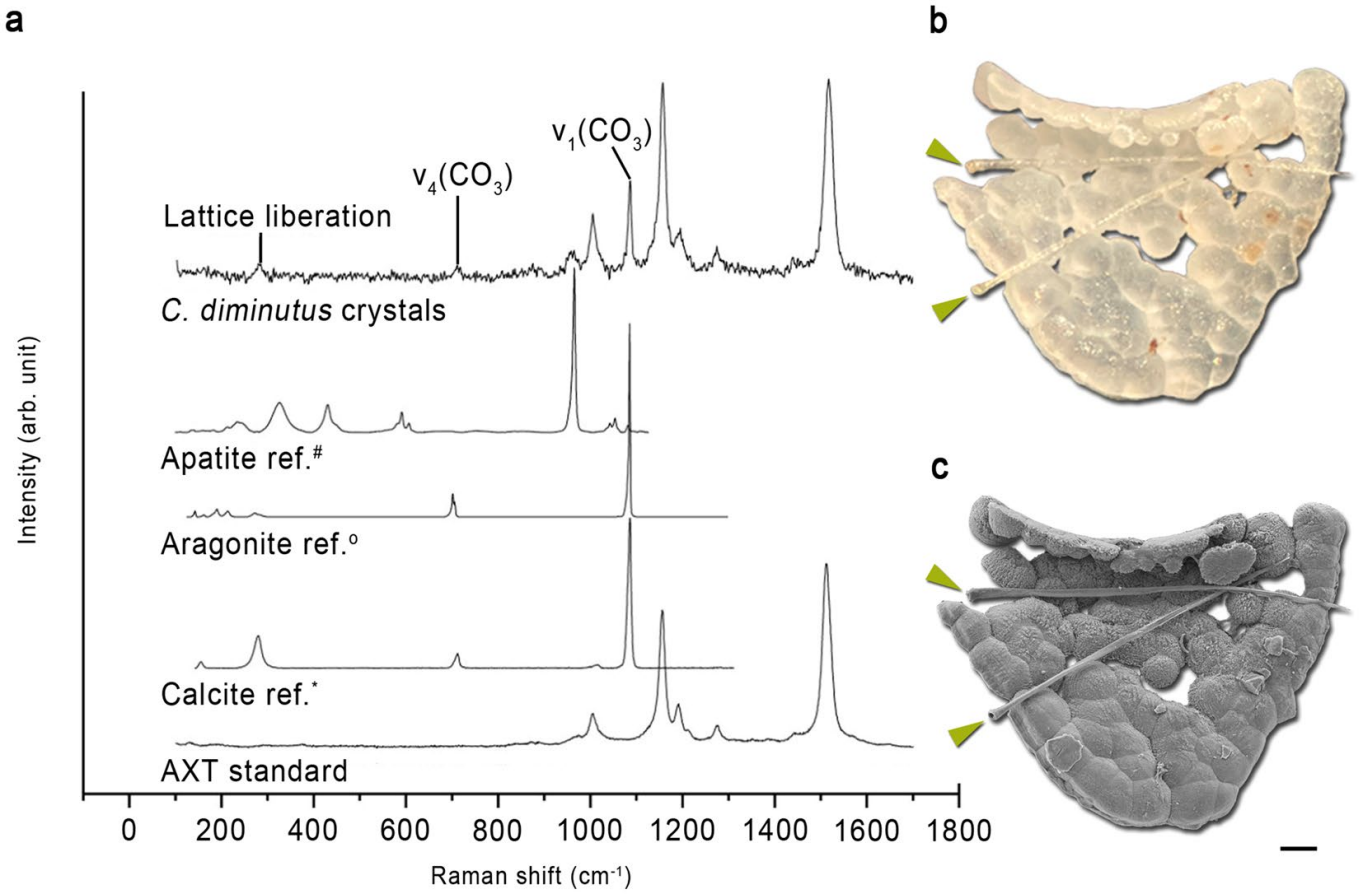
(**a**) Representative Raman spectra of observed crystal clusters compared to Raman reference spectra of crystalline apatite, aragonite and calcite, taken from the RRUFF Raman data base (^#^R060070, ^°^R060070, *R040170^24^). Raman spectra of the crystal clusters exhibit main Raman bands typically for crystallized calcite and the β-carotene, astaxanthin. (**b**) A stereomicroscopic image of a calcite conglomerate of crayfish specimen C4 with two calcified setae (green arrows). (**c**) SEM-image of the cluster with two complete calcified seta (green arrows) [WD: 22.11 mm]. Scale bar 100 µm. *WD* working distance.

On day 1, specimens C1 to C7 were blue in colour and articulated, lying with the lateral bodyside on the sediment (Fig. 1a–d). The cephalothorax of each individual had changed its colouration from blue to dark red-brown on day 2. In addition, a translucent to milky translucent biofilm had formed on the carcasses and the carcasses C1, C3, C5 and C6 were twisted by about 90 degrees due to the formation of the biofilm (Fig. 1a,c). On day 3 the specimens were completely covered by the biofilm (Fig. 1a–d). An accumulation of putrefaction gas could be noticed around the branchial area of specimens C1 and C5, which resulted in a floating of the carcasses inside the biofilm, however, the biofilms held the carcasses to the ground (Fig. 1a). A gas accumulation could also be noticed in specimens C2, C6 and C7 but was not sufficient to let the carcasses “float” inside the biofilm. On day 4 the carcasses C1 and C5 had risen further within the biofilm, but were still fixed to the sediment. From day 5 to day 7 the gas accumulation increased and, in all specimens, the abdominal muscles had changed their colouration from white to pink. On day 7, ostracods had populated the carcasses and had started to degrade the biofilms. Most of the biofilms were degraded on day 8 and ostracods started to feed on the carcasses. On day 9 nearly the complete inner organs of the carcasses had been consumed by the ostracods. In all specimens the complete hepatopancreases remained, which were hard and crumbly (Fig. 2a). In addition, crystal clusters were found in all carcasses (Fig. 3).

#### Experiment 2

On day 1, specimens C8 to C10 were blue in colour and articulated, with the left half of the body lying on the sediment. The cephalothorax of the individuals had changed their colour from blue to red on day 2, and specimen C9 was floating at the water surface, this was probably caused by putrefaction gas, which accumulated at the branchial area. On day 3, a light white biofilm could be noticed around the cephalothorax of specimen C8 (Fig. 1e) and around the pleon of specimens C9 and C10, both lying on the ground. The abdominal muscles of each specimen had changed their colouration from white to pink. Cuticles of all individuals were completely red in colour and ostracods had populated the carcasses. On day 4 organs inside the cephalothorax of specimen C8 had been consumed up by the ostracods, except the hepatopancreas and the biofilms had been completely degraded (Fig. 1e). On day 5 nearly the whole carcass of specimen C8 had been consumed by the ostracods and only the chelipeds without any tissue inside as well as the hepatopancreas were left over (Fig. 1e). The anterior part of the cephalothorax of specimens C9 and C10 were degraded and gastroliths of specimen C9 were exposed. On day 8 nearly the complete carcasses of specimens C9 and C10 had been consumed the ostracods and only the hepatopancreases of both specimens and the gastroliths of specimen C9 remained. The hepatopancreas of all specimens were slightly yellow, soft and fragile.

#### Experiment 3

On day 1, specimens C11 to C13 were blue in colour and articulated, with the left half of the body lying on the sediment. With progressive decay cuticles became translucent, reddish and the muscles were pink (Fig. 1f). On day 7 internal organs had been mostly decomposed except the intestine and ganglia. Hepatopancreases could not be detected. In addition, muscles were pulpy and the cuticles of the cephalothorax and pleon were soft and jellylike. The chelipeds were still solid after 7 days. During the whole time, biofilm formation could not be detected and no gas accumulation occurred at the branchial area. The individuals were still articulated at the end of the experiment but ruptured at the transition from the cephalothorax to the pleon and the legs disarticulated quickly during the attempt to move them out of the tank. In addition, a precipitation of crystal clusters was noticed in all carcasses.

### 16S rRNA gene amplicon sequencing

**Figure 4:**
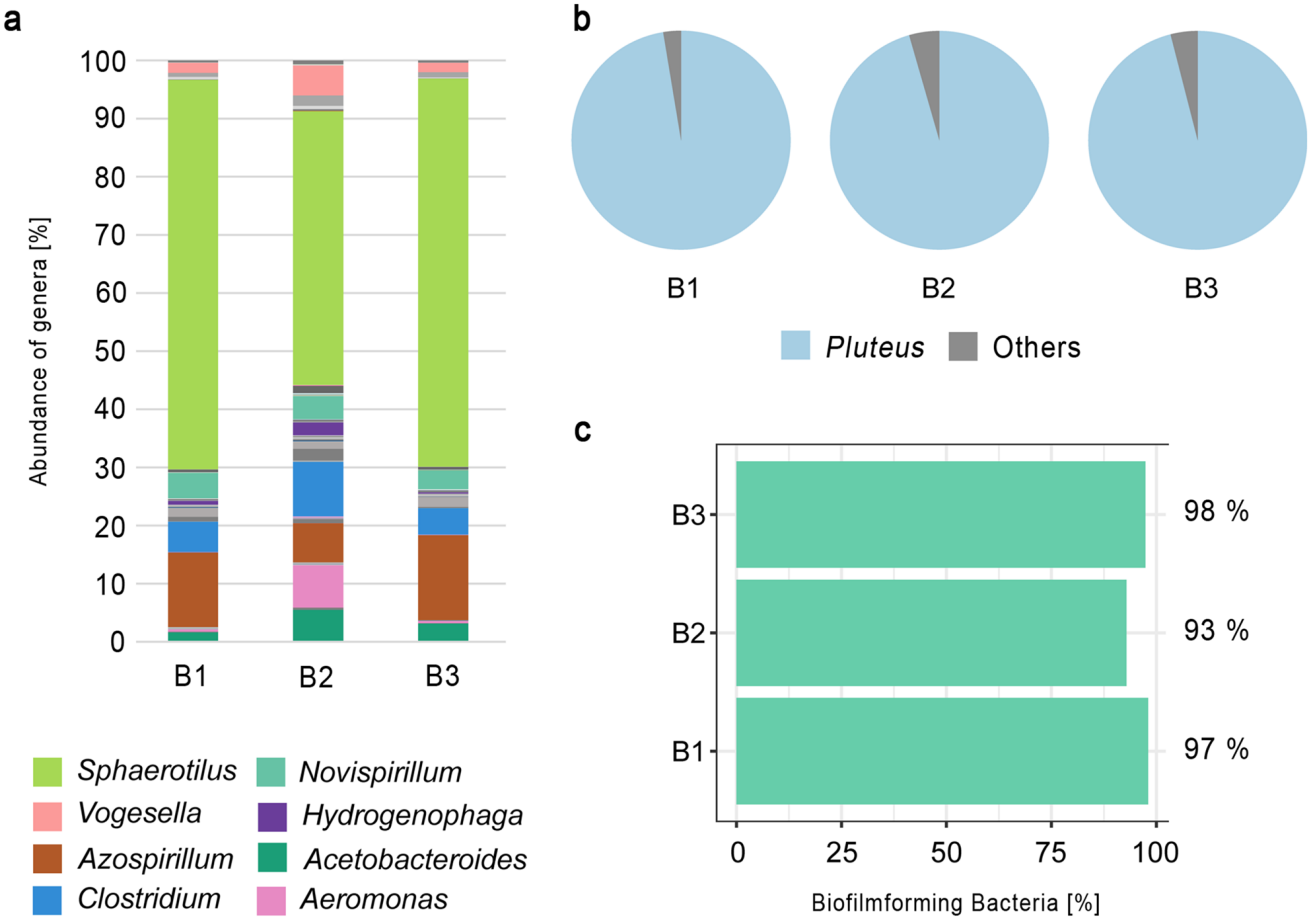
Microbial community composition of three biofilm samples taken from experiment 1 (B1 = C1; B2 = C2 and B3 = C4). (**a**) Abundance of bacterial genera (%). (**b**) Composition of fungal genera, which was dominated by the genus *Pluteus*. (**c**) Comparison of predicted biofilm forming ability of the bacteria detected in the three biofilm samples.

The 16S rRNA and ITS amplicon analyses of C1, C2 and C4 revealed that bacteria of the phyla γ-Proteobacteria, α-Proteobacteria, Bacteroidetes, and the class Clostridia were present in the biofilm. In particular, the samples were mainly composed of Gram-negative genera, such as *Sphaerotilus* [Kutzig 1833]*, Azospirillum, Hydrogenophaga,* or *Novispirillum* (Fig. 4a). In addition, the fungal colonisation was almost completely dominated by species of the genus *Pluteus* [Fries 1857] (Fig. 4b). Further on, almost all bacterial individuals exhibited biofilm forming ability in a bioinformatic analysis with the prediction tool BugBase (Fig. 4c).

### Micro-computed tomography (µ-CT)

**Figure 5:**
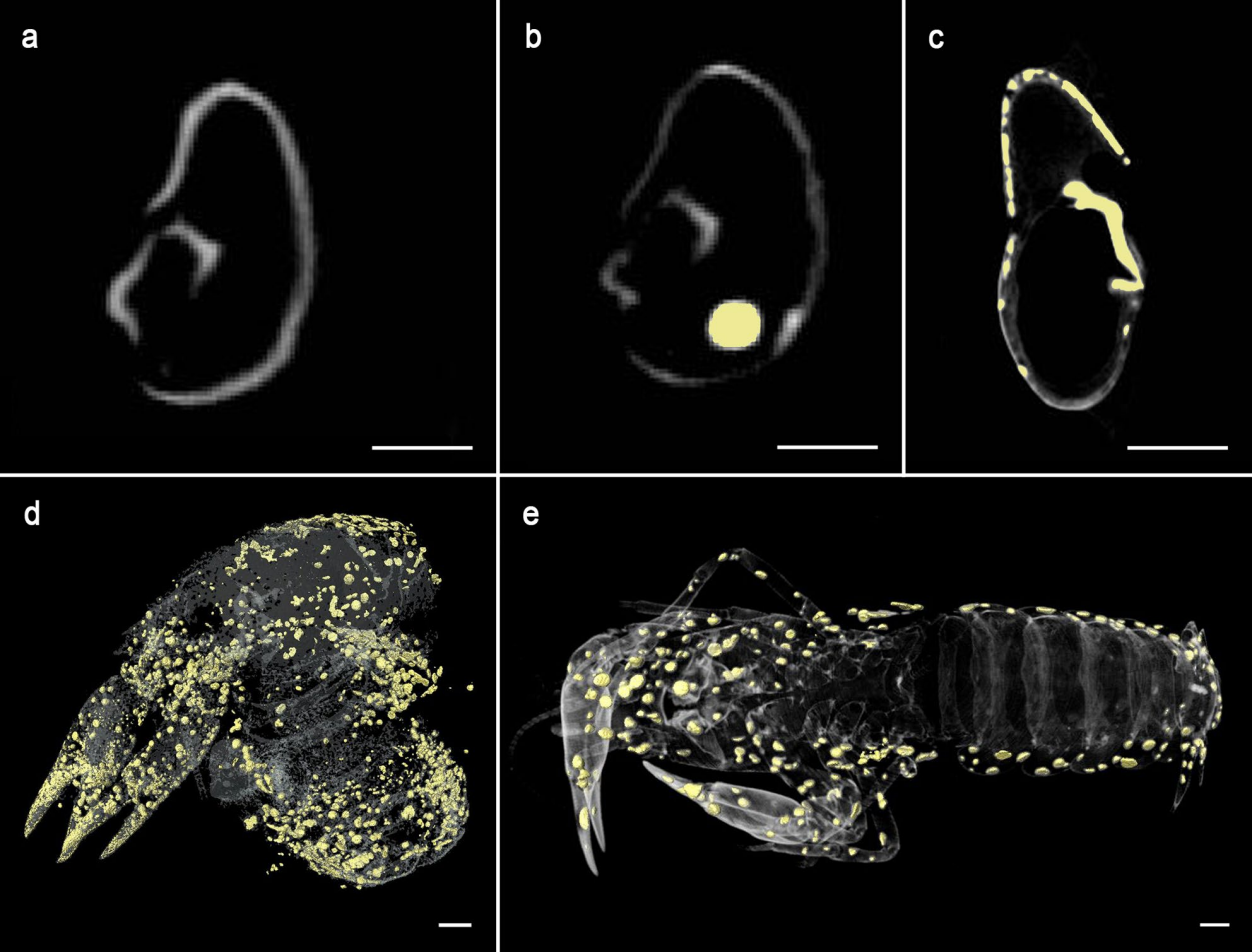
µ-CT images and 3D models of crayfish individuals and precipitated crystal clusters. (**a**) µ-CT image of a cross section of the right chela of an individual of *Cambarellus diminutus* (C7_tank_) in tank water of an experiment described in Mähler^6^ on day 1, with calcified cuticle. (**b**) The same chela as in (a) on day 7 with less calcified cuticle and a crystal cluster (yellow spot). (**c**) µ-CT image of a cross section of the left chela of C4 on day 9 with recrystallised cuticle (yellow structures). (**d**) Translucent 3D-model of C6 on day 9 with 3D-models of precipitated clusters (yellow spots). (**e**) Translucent 3D-model of an individual of *C. diminutus* (C7_tank_) in tank water of an experiment described in Mähler^6^ on day 7 with 3D-models of precipitated clusters (yellow spots). All scale bars 1 mm. µ-CT images were processed with VG Studio Max 3.2 (https://volumegraphics.com) and 3D models were reconstructed with Avizo 8.1 (https://thermofischer.com).

In contrast to coarse-grained calcite precipitations which occur in decomposing crayfish without a biofilm, µ-CT observations of the chela of specimen C4 and the complete carcasses of specimens C1, C2 and C6 revealed a precipitation of fine-grained crystal structures mostly inside the cuticle but also inside the pereiopods and chelipeds (Fig. 5a–e).

### Confocal Raman spectroscopy (CRS)

Raman spectra were obtained for an altered *C. diminutus* [Hobbs 1945] hepatopancreas (specimen C4, adipocere) and saturated fatty acid reference materials (solid stearic and palmitic acids). All strong Raman bands obtained for specimen C4 (at 890, 1062, 1098, 1128, 1295, 1440, and 1460 cm^−1^) are typical for Raman spectra of saturated fatty acids^25,26^ (Fig. 2c). In addition, there are unassigned bands at 935 and 958 cm^−1^, and a broad shoulder around ~ 1250 cm^−1^, which may point to the presence of some additional compound(s). We also observed, that none of the Raman-active bands could be detected in a fresh hepatopancreas, indicating that saturated fatty acids must have formed post mortem.

Raman analyses clearly revealed that the crystal clusters consist of well-ordered calcite (Fig. 3a), which can be identified by the fully symmetric v_1_(CO_3_) carbonate band near 1085 cm^−1^, as well as the presence of lattice vibrations near 154 and 281 cm^−1^, with the latter being absent in amorphous calcium carbonate (ACC). A mixed spectrum of crystalline calcite and the β-carotene, astaxanthin (AXT) could be identified at calcified cuticle remains on the clusters (Fig. 3a) by the typical high intensity modes at ~ 1157 and ~ 1517 cm^−1^ that are assigned to the C=C and C–C stretching vibrations of the polyene chain bonds, respectively^27–29^. In comparison to the spectrum obtained from the AXT standard, a small derivation in the frequency of the ~ 1517 cm^−1^ signal was observed. Such a shift can presumably be linked to the structural differences of AXT.

### Scanning electron microscopy (SEM)

SEM analyses of the thoracic skeleton reveal the presence of fungi inside the biofilm (Supplementary Fig. S2). Crystal clusters of C1 to C7 varying in size from ~ 100 to ~ 200 µm with the largest conglomerate measuring 1200 µm (Fig. 3b,c) at the end of the experiment. Most of the structures were spherical or bispherical (Supplementary Fig. S3a). The largest structure presented a conglomerate of layered calcite structures combined with calcite bundles and two perfectly mineralised setae found between the pulpy remains of specimen C4 (Fig. 3b,c). The dactylus of the right chela of specimen C4 showed a lot of calcite clusters instead of the original cuticle (Supplementary Fig. S4). SEM images of the hepatopancreas of specimen C4 showed in contrast to crystalline structures a pattern resembling to cauliflower (Fig. 2b).

### High performance liquid chromatography coupled to ultraviolet and mass spectrometric detection (HPLC–UV/MS)

#### Identification of free fatty acids

**Figure 6:**
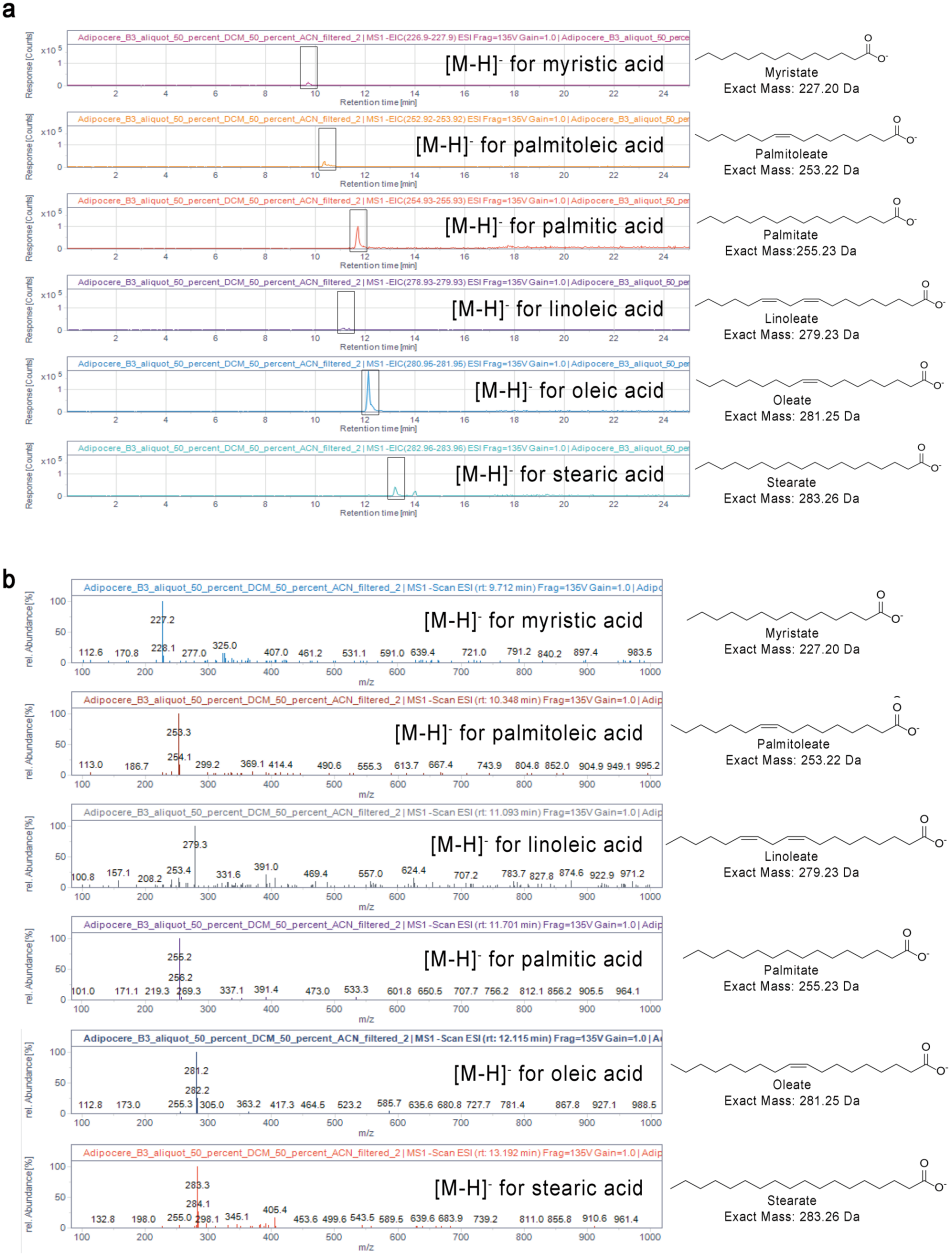
(**a**) Extracted ion chromatograms (EIC) showing the deprotonated ion of myristic acid (myristate, 227.2 ± 0.7 m/z), palmitoleic acid (palmitoleate, 253.2 ± 0.7 m/z), palmitic acid (palmitate, 255.2 ± 0.7 m/z), linoleic acid (linoleate, 279.2 ± 0.7 m/z), oleic acid (oleate, 281.3 ± 0.7 m/z) and stearic acid (stearate, 283.3 ± 0.7 m/z) in the adipocere extract, proving the presence of all of these free fatty acids in the adipocere extract. The peak areas observed in the chromatograms are shown indicating the relative amounts of acids present in the sample. (**b**) Electrospray negative ion mass spectra (ESI–MS) showing the deprotonated ions of myristic acid (myristate, 227.2 ± 0.3 m/z), palmitoleic acid (palmitoleate, 253.2 ± 0.3 m/z), palmitic acid (palmitate, 255.2 ± 0.3 m/z), linoleic acid (linoleate, 279.2 ± 0.3 m/z), oleic acid (oleate, 281.3 ± 0.3 m/z) and stearic acid (stearate, 283.3 ± 0.3 m/z) in the adipocere extract, proving the presence of these free fatty acids in the extract. The mass-to-charge ratios (m/z) are shown in relative abundance.

HPLC–MS analysis of the untreated hepatopancreas extract of specimen C4 detected six of the free fatty acids typically found in adipocere, namely palmitic acid, stearic acid, oleic acid, myristic acid, linoleic acid, and palmitoleic acid. Peaks corresponding to each of the six fatty acids were visible in the extracted ion chromatograms (EIC) at the respective mass-to-charge ratio (Fig. 6a). To confirm their identity, a standard method, also known as spiking, was used in case of oleic acid, palmitic acid and stearic acid. Here, a defined amount of the fatty acid standard was added to the sample. An increase in the peak area was observed in the extracted ion chromatograms corresponding to the spiked fatty acids (Supplementary Table S2). Furthermore, there were no new peaks detected in the extracted ion chromatograms after spiking, confirming that the increase in peak area was due to the added free fatty acid standard.

Moreover, free fatty acids originally present in the sample could be readily detected in the negative ion mode of the mass spectrometer as deprotonated ions with a mass-to-charge ratio of [M–H]^−^, where M is the monoisotopic mass of the acid (for details, see Supplementary Fig. S5 and Supplementary Fig.S6). Observation of the full scan electrospray ion (ESI)-mass spectra of the peaks (Fig. 6a) further confirmed the presence of palmitic acid, stearic acid and oleic acid in the sample. Although standard compounds for further fatty acids were not studied, myristic acid, palmitoleic acid and linoleic acid could be identified based on their mass (Fig. 6a,b).

#### Analysis of triglycerides

**Figure 7:**
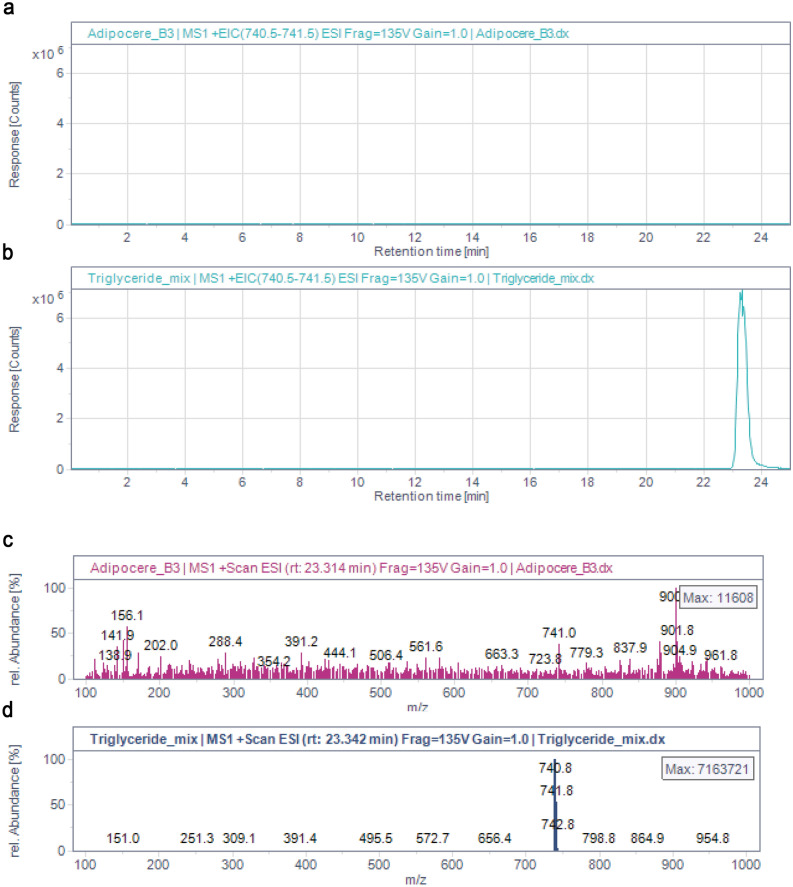
(**a**,**b**) Extracted ion chromatograms (EIC) showing the ammonium adducts (M + 18) of the glyceryl trimyristate (722.6 + 18 = 740.6 ± 0.7 m/z), in the adipocere extract (**a**) and in the standard solution containing glyceryl trimyristate (**b**). (**c**,**d**) Electrospray positive ion mass spectra (ESI–MS) showing the ammonium adduct of glyceryl trimyristate (740.6 ± 0.3 m/z) indicated that the triglyceride was not present in the adipocere extract (**c**), but only in the standard solution (**d**). The mass-to-charge ratios (m/z) are shown in relative abundance.

Since myristic acid was present in the sample, glycerol trimyristate (M = 721.6) was studied as a standard compound potentially present in the living crayfish. Mass spectra of triglycerides, determined under the applied conditions, show the ammonium adduct of the intact triglyceride in highest abundance, corresponding to M + 18 in the positive ion mode, where M is the monoisotopic mass of the triglyceride. Glyceryl trimyristate was not detected in the hepatopancreas sample, as shown in Fig. 7a–d. We only observed an unknown mass of 900.8 ± 0.3 m/z (Fig. 7c,d). The inability to detect glyceryl trimyristate or closely related triglycerides in the sample indicates that these must have been degraded. The lack of triglycerides, and the detection of free fatty acids expected to be present in adipocere confirms that the analysed hepatopancreas sample is indeed adipocere.

## Discussion

The results of our study show, the fragility of the conditions that lead to a preservation of soft tissues or their complete decomposition. Experiment 1 and 3 were conducted under the same abiotic water conditions (temperature [28 °C]; pH [8]; oxygen saturation [8 mg/L]) and the same aquatic sediment (Dehner GmbH & CoKG, 86641 Rain, Germany), and water type. However, the course of decomposition of crayfish individuals of the same species, which had been raised in the same tank community, was completely different. While in tank 1 of experiment 1 biofilm formation with an envelopment of the whole carcass occurred and gas accumulation in the branchial area could be detected, none of these occurrences could be observed in any other crayfish, which decomposed in tank 2 during experiment 3. In addition, the tissue transformation of the hepatopancreases into adipocere occurred only in individuals which were covered by a biofilm. We assume that the microbial composition inside the tanks was different and therefore extrinsic bacteria were responsible for the significant differences in decay. We assume further, that inside the biofilm oxygen was metabolised by microbial activity, resulting in anaerobic conditions which favoured the adipocere formation, since fatty acids are stabilized by these conditions^30^. Other requirements, e.g., high temperatures (28 °C) and a wet environment (tank water) were also fulfilled^31,32^. Furthermore, the genus *Clostridium* [Prazmowski 1880] was detected inside the biofilm, which is commonly associated with the formation of adipocere^33^, since these bacteria are strong hydrolysers of triglycerides. It is assumed, that the initial formation of adipocere is mainly driven by Gram-positive bacteria, whereas it is important that in the final stages Gram-negative bacteria dominate due to adipocere degradation^34,35^. The bacterial composition in and on the biofilm of individuals of experiment 1, was dominated by Gram-negative organisms and some of these might be involved in biofilm formation, e.g. *Sphaerotilus* [Kutzig 1833]. This genus contains species which settle on surfaces and form filaments that are covered by a sheath and slime^36^ (Fig. 1a, day 5). SEM-images of crayfish remains from experiment 1, that was enveloped by a biofilm show the presence of fungi that form branched mycelia (Supplementary Fig. S1) and 16S rRNA analyses of the biofilm show that the fungi genus *Pluteus* [Fries 1857] was the most abundant genus. Therefore, we assume that *Pluteus* [Fries 1857] might play an important role in our biofilm environment in experiment 1.

The genus *Pluteus* [Fries 1857] is mostly known from wood remains and food, but was also first identified by Niu^37^ as the most abundant genus in activated sludge of eastern waste water treatment plants in China. But its metabolic activity was still unknown. In 2019, Booth^38^ investigated the role of fungi in heterogenous sediment microbial networks in Mangrove sediments and found out that fungi play the major role in all microbial network interactions. They further showed, that the genus *Pluteus* [Fries 1857] [as a saprophyte (= heterotrophic organism that live in decomposing organic substances)] formed significant keystone nodes in the subsurface sediments and was one of the most important fungi genera in the microbial network^38^. Booth^38^ assumed that the fungi acting synergistically with other environmental variables and determine the overall microbial community structure. If the genus *Pluteus* [Fries 1857] was important for the biofilm formation and/or the adipocere occurrence will be investigated in further studies.

Adipocere is the result of the incomplete hydrolysis of fat in animal tissue by bacteria under mainly anaerobic conditions, because the degradation of fatty acids is restricted to respiratory processes^10^. Under anaerobic conditions fatty acids cannot undergo β-oxidation and are degraded only very slowly.

Caused by the ability of adipocere to slow down or inhibit decay processes^11^ it has been suggested as a key component in the outstanding preservation of fossils in Konservat-Lagerstätten like Messel, Holzmaden^12^, or Solnhofen^13^. It is also assumed, that adipocere preceded the phosphatization of insects discovered from Quercy (France), as a shaping component^14^.

In 2020, the crustacean-like specimens of the arthropod *Dollocaris ingens* [Van Straelen 1924], found in the Jurassic Konservat-Lagerstätte of La Voulte-sur-Rhône (France), were reinvestigated to clarify their preservation pathway^9^. These fossils show an exceptional morphological preservation of inner structures (e.g., muscles and hepatopancreas), which were preserved by fluorapatite and pyrite. Here, the transformation of the inner structures must have happened rapidly post mortem, when the sediment was still moist, loose and not complete anaerobic^9^. Jauvion^9^, as well as Wilby^39^ state, that the fossilisation process occurred simultaneously with the biodegradation and was influenced by the tissue type and local microenvironments. This assumption is supported by the results of Grimes^40^, which show that the pyritisation of plant cells depends on the plant type and the specific conditions. In addition, Jauvion^9^ did not exclude the possibility of a precipitation of pyrite and/or fluorapatite due to a biofilm.

In view of the fact, that the anatomical structure of the hepatopancreas of *Dollocaris ingens* [Van Straelen 1924] was very similar to that of modern crustaceans^41^, and based on the fast transformation of the hepatopancreas into adipocere (only in 9 days) in experiment 1 of this study, we assume that the hepatopancreases of *Dollocaris ingens* [Van Straelen 1924] might also first have been stabilised by adipocere before they were preserved in pyrite. We are aware that the here presented results are based on freshwater processes, but the formation of adipocere is also known from decomposing human bodies or pigs in marine environments^42,43^. Further on, Grimes^40^ published that the pyritisation did not directly replace the original tissue. The fossilisation was a result of precipitation of crystals on and between cells resulting in filling out of extracellular spaces. Grimes^40^ hypothesized that, as microbial decay continued, more space would become available for pyrite crystals resulting in a cast of the original material. For *Dollocaris ingens* [Van Straelen 1924] it is conceivable that during the decay under partly aerobic conditions triglycerides were hydrolytically split into glycerol and fatty acids and the sulfur-containing amino acids (cysteine and methionine) were degraded, whereby sulfur of the sulfide group was oxidised to sulfate or released as hydrogen sulfide deeper in the tissue^44^. In the anaerobic environment the fatty acids were degraded very slowly resulting in the formation of adipocere. However, over the years, bacterial syntrophic communities or bacterial species that are able to perform both reactions, use sulfate as alternative electron acceptors and degrade fatty acids (*Desulfobacteriaceae*, *Desulfarculaceae*, *Desulfohalobiaceae*, *Syntrophobacteraceae*, and *Peptococcaceae*)^45–47^, degraded the adipocere and released hydrogen sulfide. From the field of forensic science, it is known that hydrogen sulfide is able to react abiotically with iron from the haemoglobin to form iron sulfide^48^, which might later react to pyrite^49^. In *Dollocaris ingens* [Van Straelen 1924] hydrogen sulfide might have reacted with the iron from the haemolymph and/or the surrounding medium. It must be investigated, whether the increase of the iron content inside the haemolymph of decapods during the moulting process^50^ might have a positive effect on the formation of iron sulfide and later the formation of pyrite.

The adipocere theory might also be interesting for the preservation of neural tissues in the arthropods of *Fuxianhuia* [Hou 1987] from the early Cambrian Chengjiang Lagerstätte in southwest China. We assume that the organic macromolecules of the central nerve system, where the tissue is enriched in lipids^51^, were first stabilised by adipocere. Adipocere can be formed out of muscle tissue, fat and sphingosine^44^. Sphingosine is a carbon rich amino acid (C-18) which forms the primary part of sphingolipids in the membrane of myelin sheaths that surround nerve cell axons^52^.

The results of our study further show a precipitation of calcite clusters inside the specimens. During the decomposition process calcium ions are dissolved out of the cuticle layers due to acidic conditions in and around a carcass caused by autolytic enzymes^6^. With progressive decay conditions become alkaline due to microbial activity and calcium ions together with carbonate ions precipitate as calcite at the inner side of the cuticles^6^. Our results show that a biofilm might influence the type of calcite precipitation. Calcite clusters appeared coarse-grained with a size of 260 to 470 µm in the absence of biofilms^6^, and appeared fine grained (100–200 µm) if a biofilm was present (Fig. 4). Further on, the biofilms of our study were able to prevent the crayfish carcasses from floating if a gas accumulation occurred, but were not able to protect them against the degradation ability of ostracods.

## Conclusions

It seems that the preservation of *Cambarellus diminutus* [Hobbs 1945] soft tissue or its complete decomposition was mainly influenced by the extrinsic microbial community of the tank water in our experiments. The hepatopancreas of our crayfish individuals were completely transformed into adipocere only in the presence of a biofilm. The biofilm was mainly composed of the bacterial genus *Sphaerotilus* [Kutzig 1833] and the fungi genus *Pluteus* [Fries 1857]. We assume that the combination of these microbial genera might play an important role in soft tissue preservation. The analyses of the altered hepatopancreas sample revealed that it contains a mixture of saturated (palmitic, stearic, and myristic acids) and unsaturated fatty acids (oleic, linoleic, and palmitoleic acids). The inability to detect glyceryl trimyristate or similar triglycerides in the sample indicates that most triglycerides have been degraded, which is typical for adipocere. We assume that, because of the early diagenetic transformation of soft tissue into adipocere (9 days) and the shape-retaining ability of this substance, adipocere might be a first step in soft tissue preservation under certain conditions.

## Supplementary Information

Supplementary Information.

## Data Availability

All raw sequence data related to this study are deposited in the European Nucleotide Archive (ENA) (European Bioinformatics Institute, EMBL-EBI) database a collaboration partner of the International Nucleotide Sequence Database (INSDC), [Study-Accession Number: PRJEB43756].
