# Supplementary material for: Adipocere formation in biofilms as a first step in soft tissue preservation
Source: Sci Rep. 2022 Jun 16;12:10122. doi: 10.1038/s41598-022-14119-8 (PMC9203803; doi:10.1038/s41598-022-14119-8)
Supplement: Supplementary file 1 — Supplementary Information. [file 41598_2022_14119_MOESM1_ESM.pdf]

Supplementary Information:

Adipocere formation in biofilms as a first step in soft tissue preservation

Bastian Mähler[1*], Kathrin Janssen[2], Mariam Tahoun[3], Frank Tomaschek[4], Rico Schellhorn[1], Christa E. Müller[3], Gabriele Bierbaum[2] & Jes Rust[1]

[1]Section Palaeontology, Institute of Geosciences, Rheinische Friedrich-Wilhelms Universität Bonn, 53115 Bonn, Germany

[2]Institute of Medical Microbiology, Immunology and Parasitology, Medical Faculty, Rheinische Friedrich-Wilhelms Universität, 53127 Bonn, Germany

[3]Pharmazeutisches Institut, Pharmazeutische & Medizinische Chemie, Rheinische Friedrich-Wilhelms-Universität Bonn, 53121 Bonn, Germany

[4]Section Geochemistry, Institute of Geosciences, Rheinische Friedrich-Wilhelms-Universität Bonn, 53115 Bonn, Germany

* Corresponding author: bastian.maehler@uni-bonn.de

Supplement

Product information "Crab Natural" (Sera, GmbH, Heinsberg, Germany)

Complete feed for all crustaceans

Ingredients

fish meal, corn starch, wheat flour, spirulina, brewers yeast, wheat germ, gammarus, Ca-caseinate, sea algae, stinging nettle, willow bark, alder cones, fish oil (containing 49% omega fatty acids), mannan oligosaccharides, herbs, alfalfa, parsley, paprika, green-lipped mussel, spinach, carrots, *Haematococcus* algae, garlic.

Analytical constituents

Crude Protein 36.7%, Crude Fat 11.1%, Crude Fiber 4.5%, Moisture 5.2%, Crude Ash 8.6%, Ca 1.9%, P 1.0%.

Additives

Vitamins and provitamins: Vit. A 37,000 IU/kg, Vit. D3 1,800 IU/kg, Vit. E (D, L-α-tocopheryl acetate) 120 mg/kg, Vit. B1 35 mg/kg, Vit. B2 90 mg/kg, stab. Vit. C (L-ascorbyl monophosphate) 550 mg/kg.

Supplementary Table S1 Information on the crayfish specimens used in the study

sample	wet weight	body size	sample	wet weight	body size
C1	0.26 g	2.50 cm	C8	0.28 g	1.80 cm
C2	0.17 g	1.80 cm	C9	0.22 g	1.80 cm
C3	0.42 g	2.10 cm	C10	0.13 g	1.70 cm
C4	0.37 g	1.90 cm	C11	0.43 g	2.40 cm
C5	0.32 g	2.10 cm	C12	0.34 g	2.00 cm
C6	0.34 g	2.10 cm	C13	0.32 g	2.10 cm
C7	0.30 g	2.30 cm	-	-	-

Supplementary Table S2 Mass-to-charge ratios (m/z) of the analysed fatty acids (Mr). In bold are the m/z values used for identification of the acids as their deprotonated ions ([M-H]).

Fatty acid	M_r	$[M–H]^-$
Myristic acid	228.2 Da	**227.2 Da**
Palmitoleic acid	254.2 Da	**253.2 Da**
Palmetic acid	256.2 Da	**255.2 Da**
Linoleic acid	280.2 Da	**279.2 Da**
Oleic acid	282.3 Da	**281.3 Da**
Stearic acid	284.3 Da	**283.3 Da**

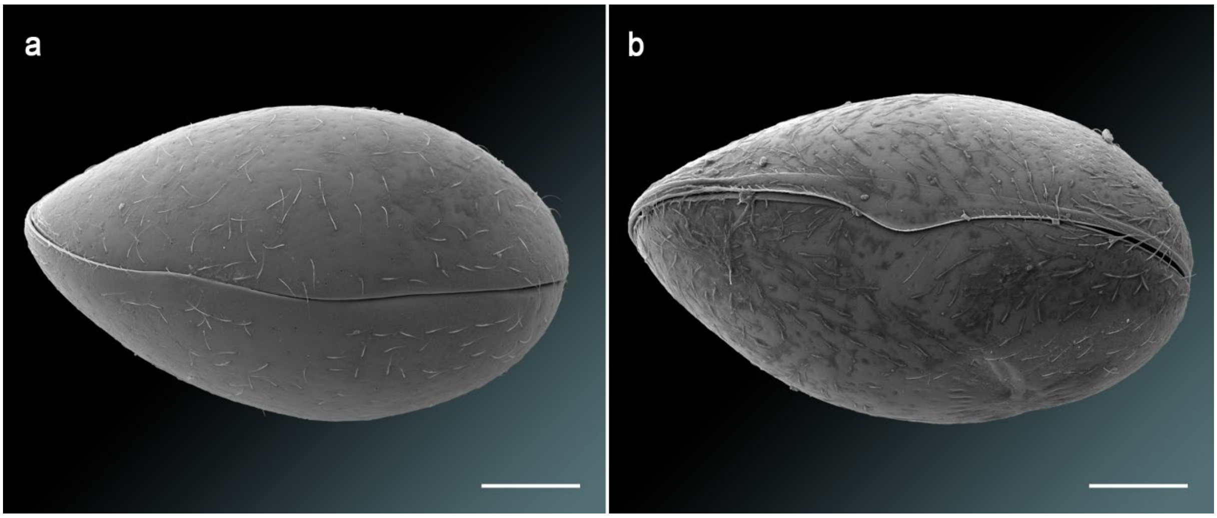

Supplementary Figure S1. (a-b) SEM-images of ostracods found on decomposing crayfish samples and biofilms [WD: 21.91 mm; 21.99 mm]. Scale bar 100 µm. **WD** working distance.

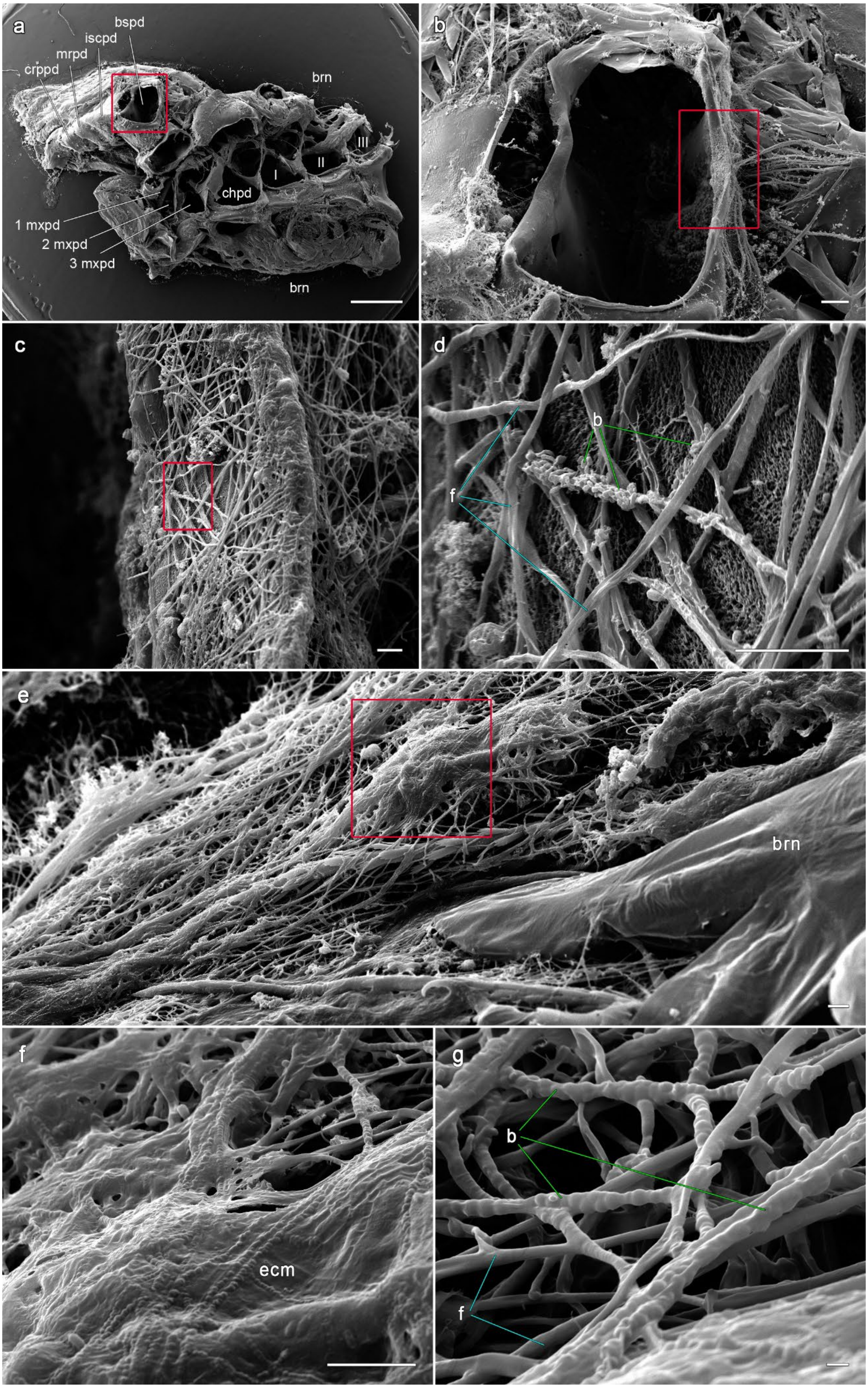
a
bspd
iscpd
mrpd
crppd
brn
III
II
I
chpd
1 mxpd
2 mxpd
3 mxpd
brn
b
c
d
b
f
e
brn
f
ecm
g
b
f

Supplementary Figure S2. SEM-images of the thoracic skeleton of a crayfish with fungal infestation. (**a**) Remains of the thoracic skeleton [WD: 9.49 mm]. Scale bar 1 mm. (**b)** Enlargement of the red box from 1a showing the right basipod [WD: 8.64 mm]. Scale bar 100 µm. (**c**) Enlargement of the red box from 1b showing the fungal infestation of the cuticle [WD: 8.62 mm]. Scale bar 10 µm. (**d**) Enlargement of the red box of 1c showing the fungal infestation with some kind of bacteria [WD: 9.63 mm]. Scale bar 10 µm. (**e**) Fungal biofilm around the branchiae. *Scale bar* 10 µm. (**f**) Enlargement of the red box from 1e showing the increase of the extracellular matrix. *Scale bar* 10 µm. (**g**) Enlargement of the fungal branches in combination of bacteria. *Scale bar* 1 µm. **WD** working distance **b** bacteria; **brn** branchia; **bspd** basipodite; **chpd** cheliped; **crppd** carpopodite; **ecm** extracellular matrix; **f** fungi **iscpd** ischiopodite; **mrpd** meropodite; **mxpd** maxilliped; **I-III** pereiopods.

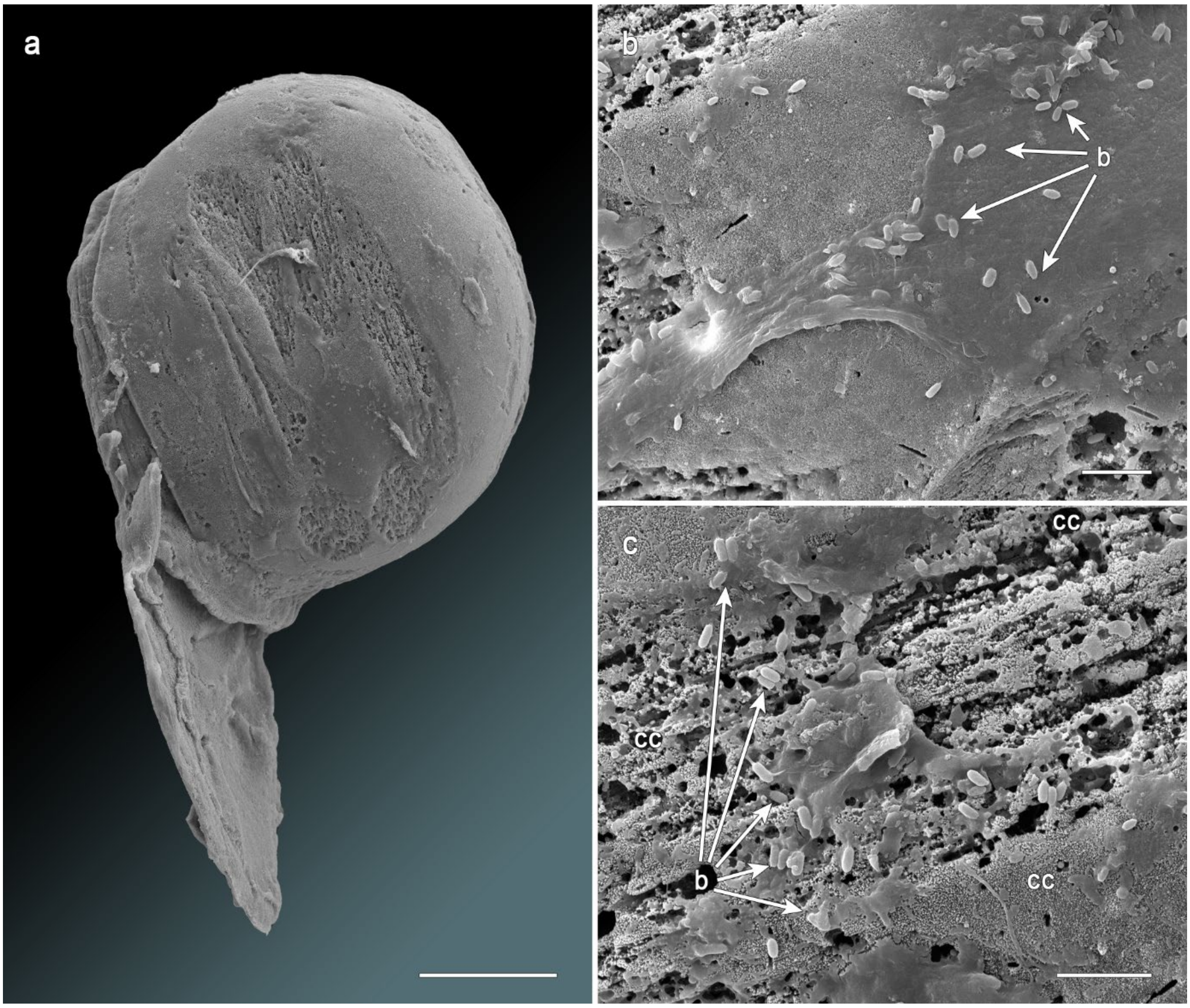

Supplementary Figure S3. SEM-images of a spherical calcite cluster. (**a**) Calcite cluster of C4 [WD: 22.14 mm]. Scale bar 100 µm (**b,c**) SEM-images of an enhanced part of the cluster with parts of a biofilm and bacteria [WD: 22.11 mm]. Scale bar 10µm. **WD** working distance.

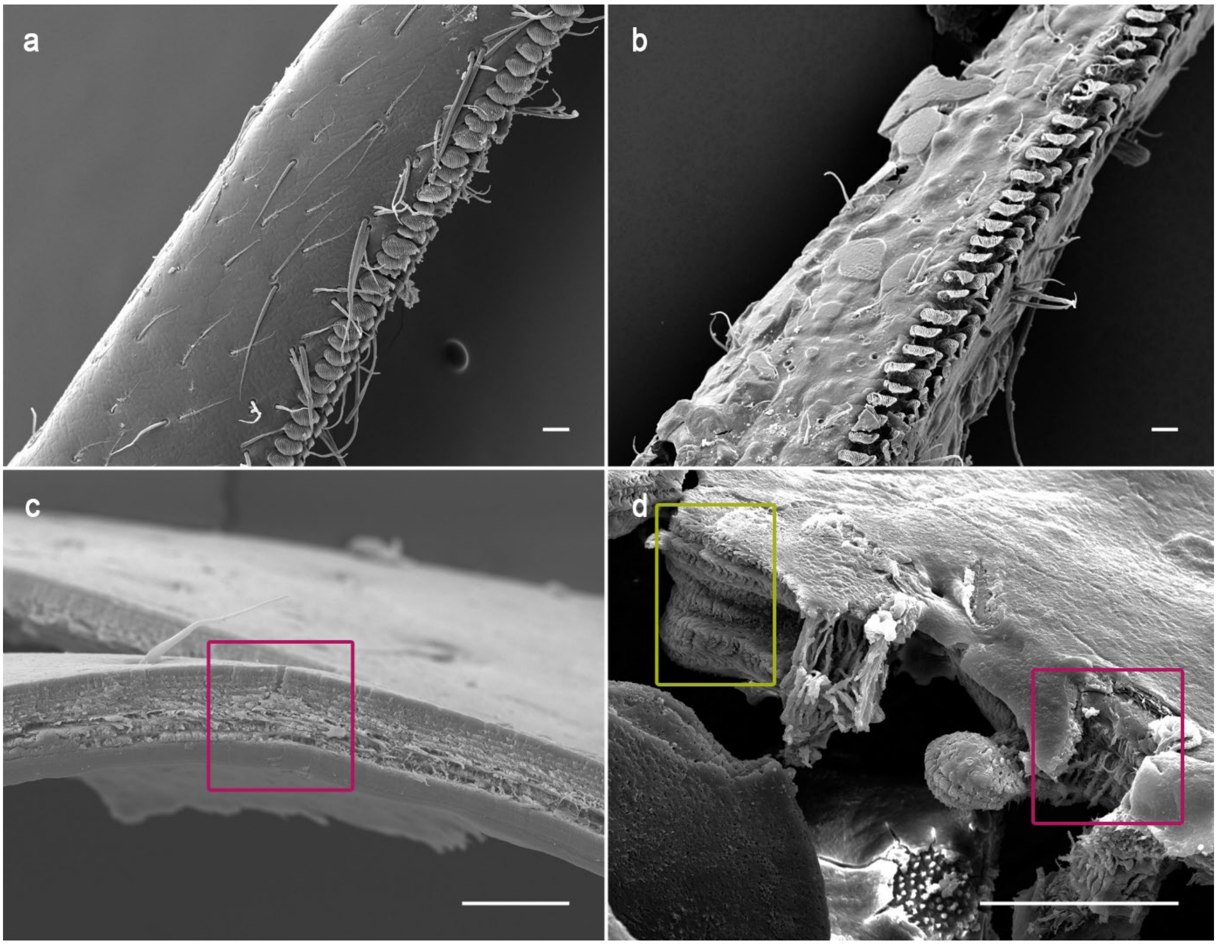

Supplementary Figure S4. SEM-images of fresh and decomposed crayfish structures. (**a**) Middle section of a fresh crayfish dactylus [WD: 21.69 mm]. (**b**) Middle section of the decomposed crayfish dactylus of sample C4 after nine days covered by a biofilm in tank water [WD: 21.03 mm]. (**c**) Cross section of a fresh crayfish cuticle [WD: 21.61 mm]. (**d**) Cross section of the cuticle of the dactylus of sample C4 showing a recrystalized part of the cuticle by calcite clusters (green box) and "unaltered" cuticle structures (pink box) [WD: 21.96 mm]. All scale bars 50 µm. **WD** working distance.

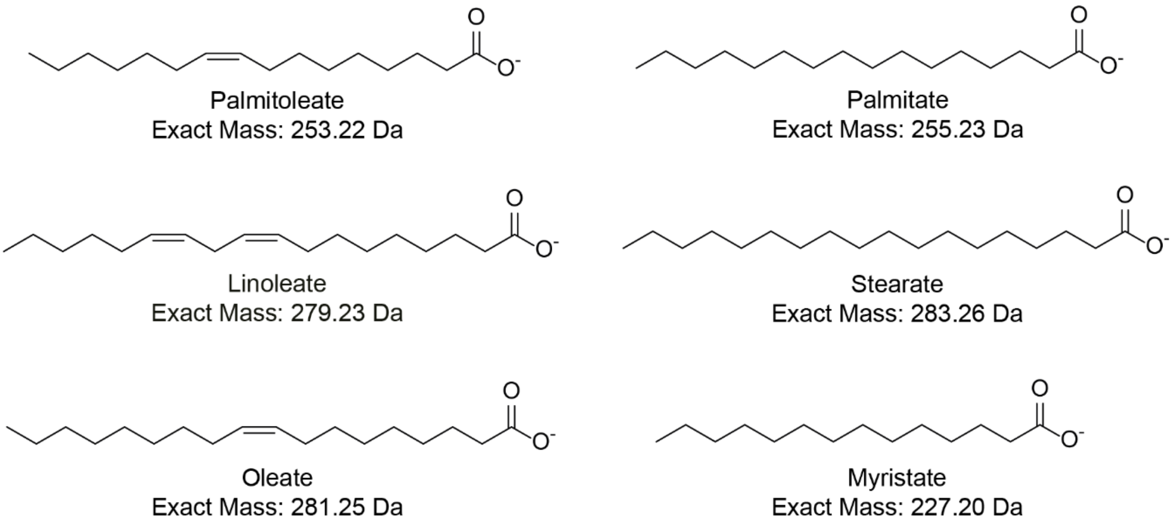

Supplementary Figure S5. Structures of the free fatty acids analysed as deprotonated species $[M-H]^-$.

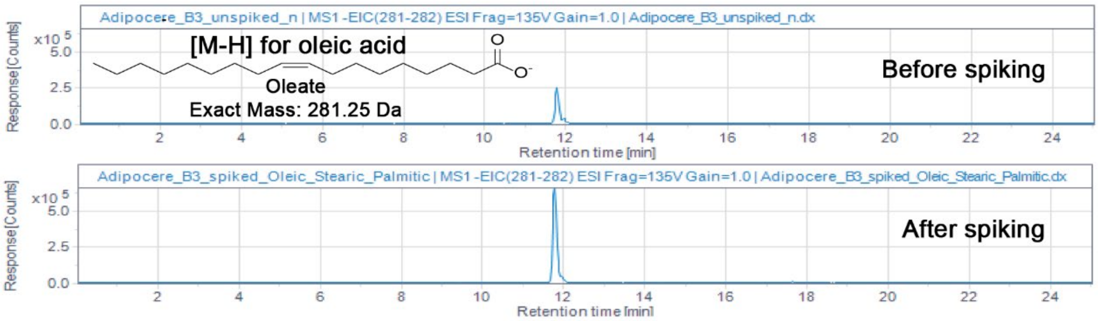

Supplementary Figure S6. Extracted ion chromatograms (EIC) showing the deprotonated ion of oleic acid (oleate, 281.3 ±0.7 m/z) in the adipocere extract, and the increase in its peak area following the addition of a known amount of oleic acid, confirming that oleic acid is present in the adipocere extract. Note that this standard addition experiment with oleic acid was performed in a different run and on a different day than the run shown in Figure 10, which explains the slight shift in retention time.
